# Supplementary material for: Pharmacogenomics decision support in the U-PGx project: Results and advice from clinical implementation across seven European countries
Source: PLoS One. 2022 Jun 8;17(6):e0268534. doi: 10.1371/journal.pone.0268534 (PMC9176797; doi:10.1371/journal.pone.0268534)
Supplement: S1 File — Requirements analysis framework and results. (PDF) [file pone.0268534.s001.pdf]

Requirements analysis

Contents

Executive summary ... 3
Introduction ... 3
Aim of this deliverable ... 3
Purpose of this document & document structure ... 3
Examples of current pharmacogenomics implementation projects ... 4
PG4KDS: St. Jude Children's Research Hospital in Memphis, USA ... 4
PREDICT: Vanderbilt University School of Medicine, Nashville, USA ... 4
CLIPMERGE PGx Program: Icahn School of Medicine at Mount Sinai, New York, USA ... 4
Personalized Medicine Program: University of Florida and Shands Hospital, USA ... 5
1200 Patients Project: University of Chicago, USA ... 5
Prospective DPYD genotyping, Leiden University Medical Center, The Netherlands ... 5
Summary ... 5
General workflow for U-PGx ... 6
Analysis of existing information infrastructures and requirements of implementation sites ... 6
Netherlands ... 6
Setting ... 6
PGx implementation workflow ... 6
IT Infrastructure ... 7
Requirements ... 8
United Kingdom ... 8
Setting ... 8
PGx implementation workflow ... 8
IT Infrastructure ... 9
Requirements ... 9
Italy ... 10
Setting ... 10
PGx implementation workflow ... 10
IT Infrastructure ... 10
Requirements ... 11
Spain ... 11
Setting ... 11

PGx implementation workflow 11
IT Infrastructure 12
Requirements 12
Austria 12
Setting 12
PGx implementation workflow 12
IT Infrastructure 13
Requirements 13
Slovenia 14
Setting 14
PGx implementation workflow 14
IT Infrastructure 14
Requirements 15
Web- and paper-based reminders for identifying eligible patients are required.Greece 15
Setting 15
PGx implementation workflow 15
IT Infrastructure 16
Requirements 16
Summary 16
Abbreviations 21
List of tables 21
List of figures 21
References 21

Executive summary

This report provides an analysis of existing health IT infrastructures and clinical workflows at the 7 different U-PGx implementation sites and aims at identifying the basic requirements that need to be met to guarantee a successful integration of pre-emptive pharmacogenomic testing at each site.

The results reveal large differences in the existing infrastructures and IT capabilities, ranging from the practical non-existence of an Electronic Health Record (EHR) and Laboratory Information Management System (LIMS) infrastructure (Greece) to sophisticated infrastructures with a full-fledged integration of active pharmacogenomics clinical decision support (CDS) alerts (Netherlands).

This report finds that, for U-PGx, providing active alerts with PGx recommendations via the EHR will only be feasible in the Netherlands. All other clinical implementation sites will require an automatic generation of a digital PGx report, which is then either transferred to the EHR or printed out and archived in the patient's paper-based record.

Furthermore, likely more than one method will be used to make the patients' PGx results available to the physicians and pharmacists at all of the 7 implementation sites. Most sites with the capability to store a digital PGx report in the EHR will also use paper-based reports. Additionally, if compatible with the legal and regulatory conditions of each country, a complementary mobile-based system for providing PGx CDS (Medication Safety Code System) will be implemented to facilitate the transfer of PGx results to external health care professionals.

Similarly, using active alerts via the EHR for reminding physicians and pharmacists that a patient is eligible for inclusion into the study will only be feasible in the Netherlands, and potentially in Spain. All other sites will require web- or paper-based reminder tools. Furthermore, in the UK, in the Netherlands an in Spain, screening the prescription list can be used as a method to identify eligible patients.

Introduction

Aim of this deliverable

To ensure the successful implementation of pre-emptive pharmacogenomic (PGx) testing at all participating clinical sites during the first year of U-PGx, it is crucial to consider the various health IT infrastructures and potentially diverging legal and regulatory conditions in each of the 7 participating countries. The aim of this deliverable is to analyze existing workflows and information infrastructures at each of the 7 implementation sites, and to provide an overview of the basic requirements that need to be met to enable an efficient integration of pre-emptive PGx testing into these workflows and infrastructures.

Purpose of this document & document structure

This document is intended to guide the implementation of the IT components that are necessary to enable pre-emptive PGx testing at the 7 different U-PGx implementation sites.

Firstly, this document will provide an overview of existing PGx implementation projects, and summarize commonalities and key points of these projects. The second part of the document concentrates on describing the current state and available infrastructure at each implementation site, and gives a concise summary of the basic requirements of each site.

Examples of current pharmacogenomics implementation projects

PG4KDS: St. Jude Children's Research Hospital in Memphis, USA

Hoffman et al. describe the clinical implementation of a pre-emptive PGx testing workflow in St. Jude Children's Research Hospital in Memphis, USA. (1) The St. Jude Children's Research Hospital focuses on providing in- and outpatient care for children with catastrophic diseases, such as cancer.

A standardized process for genotyping, interpreting and transferring the results to the EHR, and for providing PGx CDS is defined in the PG4KDS protocol. After array-based genotyping is done in an associated CLIA laboratory, a diplotype translation report is sent to St. Jude via Secure File Transfer Protocol. PGx test results are then processed through two in-house custom web-based applications (DMET Tracker and Consult Builder), and incorporated into the EHR to enable active CDS. The DMET Tracker software is used to review and check the PGx test results, which is done by an informaticist and a pharmacist. The Consult Builder application matches the results with predefined consults (i.e. phenotype assignment, interpretation of the diplotype, dosing recommendations, activity score where applicable).

Every consult is manually reviewed by a second pharmacist, and patient-specific comments are added if deemed necessary. Patients are informed of their PGx testing results and potential implications for their medication via a letter. Furthermore, diplotype results are stored in a separate research database.

PREDICT: Vanderbilt University School of Medicine, Nashville, USA

The PREDICT program, established in September 2010 at the Vanderbilt University School of Medicine, aims at implementing pre-emptive genotyping for high-impact genetic variants. The project initially focused on CYP2C19 genotyping of patients scheduled for coronary arteriography. (2) Genotyping results are stored in a secure database separate from the EHR, and are incrementally released to the EHR every time a new genotype is deemed actionable by an expert committee. Actionable genotypes are then converted into a standard notation including a phenotype interpretation and stored in the EHR as a molecular diagnostic lab result. PGx results are displayed in the EHR in a "Drug-Genome Interaction" section of the patient summary page. Active CDS is integrated within inpatient and outpatient electronic prescribing application.

CLIPMERGE PGx Program: Icahn School of Medicine at Mount Sinai, New York, USA

Gottesman et al. describe the implementation of a PGx program (CLIPMERGE) at the Icahn School of Medicine at Mount Sinai in New York. (3) For the study that started in February 2013, a pilot cohort of patients who had regularly attended the Internal Medicine Associates over the past two years where selected from an existing DNA biobank. Furthermore, patients who were likely to receive certain medications with known PGx interactions (e.g. clopidogrel, warfarin, etc.) were included into the study.

Saliva samples are collected from each participating patient, and genotyping is performed at the CLIA-certified Mount Sinai Genetic Testing Laboratory. Genotyping results are transferred to a data management system that is external to, but communicates with the local EHR. Furthermore, longitudinal clinical data are fed back from the EHR to the CLIPMERGE data management system. A Clinical Risk Assessment Engine (CRAE) includes a rule engine for matching genotypes to relevant

clinical PGx recommendations. Relevant recommendations are made available as active alerts at the point of care through the local EHR.

Personalized Medicine Program: University of Florida and Shands Hospital, USA

In 2011 the University of Florida and Shands Hospital established a pre-emptive PGx testing program, called "Personalized Medicine Program". (4) For the program, a custom and easily adaptable chip covering 256 drug-metabolizing enzyme and transporter SNPs with sound evidence of clinical utility was designed. (5) The decision to move PGx results to the EHR is made by an expert committee upon evaluating the literature, and formulating the recommendation for incorporation into the CDS tools to enable active CDS at the time of prescribing. The project initially focused on CYP2C19 genotyping for patients getting left heart catheterization. At the University of Florida and Shands Hospital, CYP2C19 genotyping is validated as a standard care procedure, and therefore no clinical consent is required.

1200 Patients Project: University of Chicago, USA

The 1200 Patients Project was established at the University of Chicago in 2011 with the aim of prospectively genotyping 1200 adults receiving outpatient medical care. (6,7) Blood samples are genotyped using a commercial panel (Sequenom ADME panel) and an additional custom-designed panel. Genotypes that are deemed actionable are classified based on their clinical implications, are assigned a color (green, yellow, red) or symbol (sideways arrow, dose-calculator), and are then transferred to the Genomic Prescribing System (GPS), a web-based portal that was specifically designed for this project. In the initial phase of the project, the patients' PGx results and dosing recommendations are accessible only to a small number of physicians (n=6) who have received special training in using the GPS.

Prospective DPYD genotyping, Leiden University Medical Center, The Netherlands

At the Leiden University Medical Center (LUMC) a pre-emptive DPYD screening program was established for all patients with an indication for a fluoropyrimidine containing therapy. (8) Samples were genotyped three-times per week using a commercial assay (TaqMan® Genotyping SNP assay) and an additional home-brew pyrosequencing method (9). Genotyping results were then made available to physicians and pharmacists via the EHR by the responsible pharmacist. Initially, patients were only screened for one DPYD variant; later on two further DPYD variants were added to the screening. A clinical decision support system (ADEAS, adverse drug event alerting system) that is connected to several other hospital information systems, including a database that contains pharmacogenomic dosing guidelines, is already part of routine care at LUMC, and was used to alert pharmacists that a patient is at risk of an adverse drug event.

Summary

Across the described PGx implementation projects, several commonalities can be identified:

(1) EHRs with the ability to generate active alerts are available at almost all implementation sites. Only one project provided PGx results and recommendations via a web-based portal separate from the EHR. However, this portal was initially only accessible by a small number of physicians who received special training in using the portal.

(2) Most implementation programs use multi-gene arrays, but initially focus on a small number of gene-drug combinations for actual clinical implementation.

(3) Raw PGx results are commonly stored in an external database that communicates with the EHR.
(4) Identification and approval for the transfer of new gene-drug combinations into the patients' EHR are systematic procedures and are often guided by an expert committee.
(5) Individual PGx test results and recommendations are often subject to manual review and approval by professionals.

General workflow for U-PGx

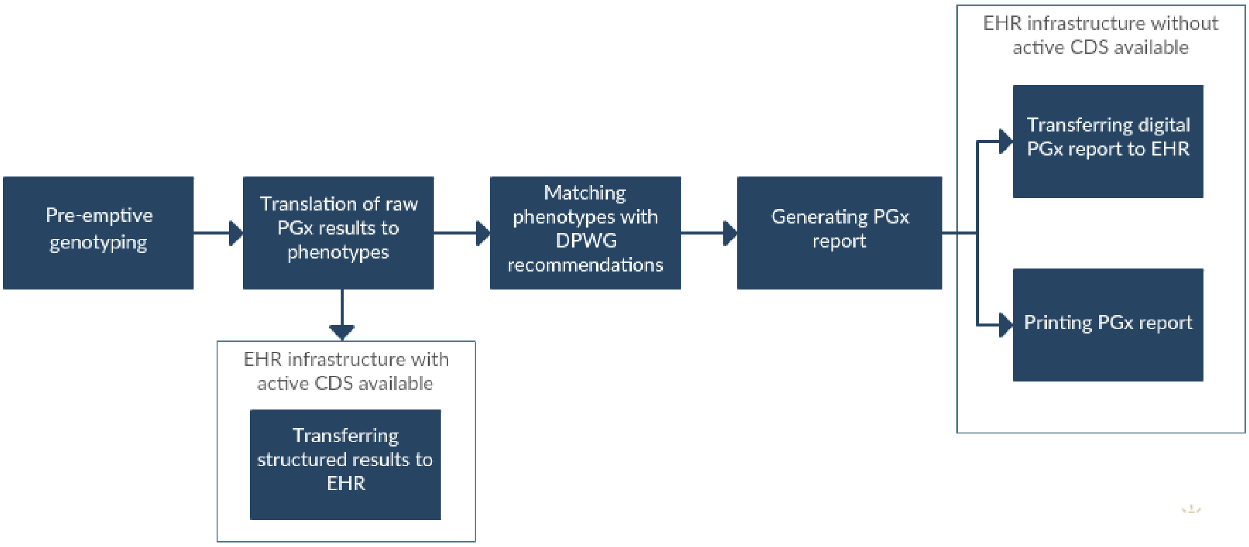

Figure 1: Illustration of the basic workflow for U-PGx.

Raw genotyping results will be translated to phenotypes using a standardized process across all implementation sites. Phenotypes will be matched with translated versions of the PGx-based drug dosing recommendations authored by the Dutch Pharmacogenetics Working Group (DPWG). The way in which PGx results are delivered to healthcare providers will depend on the available IT infrastructure at the implementation site.

Analysis of existing information infrastructures and requirements of implementation sites

Netherlands

Setting

In the Netherlands, pre-emptive PGx testing will be implemented in a network of primary care physicians and pharmacies established by the department of Clinical Pharmacy & Toxicology of the Leiden University Medical Center (LUMC).

PGx implementation workflow

1. Identifying patient for PGx testing eligibility

Currently, two methods for identifying patients eligible for PGx testing are used in the **outpatient setting**, depending on the available IT infrastructure:

- Half of pharmacists are reminded by active alerts via the tool "N-Control" whenever a patient is about to receive a relevant PGx medication.

- The other half of pharmacists do not have active alerts available. Therefore, they screen the list of patients at the end of the day and re-contact those patients who have received a relevant PGx medication.

In the **hospital setting**, no workflow for identifying eligible patients has yet been established at LUMC. Establishing a suitable workflow for this step—ideally the implementation of active alerts whenever a relevant PGx medication is prescribed—will be promoted within the context of U-PGx.

2. *Ordering PGx test*

The current practice for ordering a PGx test is as follows: If a patient is identified to be eligible for PGx testing, he is contacted by the physician. The patient either comes back to provide a saliva sample, or the test tube is sent via mail. In the latter case, the patient mails the saliva sample back to the hospital which, however, may cause the PGx testing process to take about two weeks.

3. *Conducting PGx test*

At the moment, PGx testing is conducted at the lab within the pharmacy. For U-PGx, PGx testing will be performed using a standardized genotyping platform provided by bio.logis.

4. *Storage of raw PGx data*

Raw PGx results are stored within the protected network environment of LUMC.

5. *Interpretation of raw PGx data*

Currently, the interpretation of raw PGx data is a manual step at LUMC. For U-PGx, the translation from raw PGx data to phenotypes will be performed by the software that comes with the genotyping platform selected by bio.logis.

6. *Storage of interpreted PGx data (=phenotypes)*

The curated results are stored in GLIMS, the lab information management system. From GLIMS, the data is transferred to HIX, the electronic medical record.

7. *Generating PGx report and transferring it to intended users*

In the Netherlands, systems for drug prescribing and dispensing have the possibility to record the patient's genotype and then automatically provide a pop-up in case of an actionable gene-drug interaction. However, there is no standardized way of transferring the PGx results outside of the hospital. At LUMC a letter is sent to the health care professional that requested the test. The MSC system would be of interest to facilitate the information transfer.

IT Infrastructure

1. *LIMS*

See *Table 2* for LIMS characteristics.

2. EHR

The EHR (HIX) has the capability to generate active alerts by using the information provided by the G-Standaard (see below).

3. G-Standaard:

In the G-Standaard DPWG recommendations are linked to the hierarchical structure of the identification of medicines (packaged medicinal products, trade products, generic [ISO standard 11616]) and geno-/phenotypes.

Requirements

- The raw PGx results provided by the genotyping platform need to be fed into a database of the protected network environment of LUMC.
- The interpreted PGx results (haplotypes, phenotypes) provided by the bio.logis system need to be fed into the GLIMS.
- From the GLIMS, the interpreted PGx results need to be transferred to the EMR (HIX).
- A way to generate and print the MSC cards based on the interpreted PGx results (haplotypes, phenotypes) needs to be established.
- (A way to identify patients eligible for PGx testing needs to be established in the hospital setting. Ideally, the EHR will be adapted to generate alerts whenever a relevant drug substance is prescribed.)

United Kingdom

Setting

The Royal Liverpool University Hospital is a tertiary teaching centre hospital and will serve as the main site for patient recruitment; hospital inpatients will be recruited. Inpatients under the care of the clinical pharmacology team will be prioritized for recruitment, but patients under the care of other departments will also be eligible.

PGx implementation workflow

1. Identifying patient for PGx testing eligibility

Patients eligible for pharmacogenomic (PGx) testing will be identified by screening the Electronic Prescribing and Medicine Administration (EPMA) ICT system for inpatients who have been prescribed any one of the U-PGx listed medications. Additional searches within EPMA will be used in potentially eligible patients to determine whether this is a new EPMA prescription or not. EPMA only includes prescriptions from inpatient episodes at the Royal Liverpool hospital; it does not cover outpatient prescriptions or regular primary care prescriptions. Therefore, during patient recruitment, patients will be asked about their recent (outpatient) medication use, and if necessary their General Practitioner (GP) will be contacted.

2. Ordering PGx test

Currently, PGx testing (e.g. *HLA-B*57:01*) is ordered electronically via the ICE desktop ICT system. This will be implemented for U-PGx.

3. *Conducting PGx test*

For U-PGx, PGx testing will be performed using a standardized genotyping platform provided by bio.logis.

4. *Storage of raw PGx data*

For U-PGx a new database will be created for the storage of raw PGx results.

5. *Interpretation of raw PGx data*

The interpretation of raw PGx data is conducted manually at present. For U-PGx, the bio.logis system will be used. Relevant anonymous data will be transferred to bio.logis for interpretation and receipt of the individualized report.

6. *Storage of interpreted PGx data (=phenotypes)*

See point 7.

7. *Generating PGx report and transferring it to intended users*

The generated PGx report is transferred to the ICE desktop system, for review by the patient's healthcare professionals.

IT Infrastructure

1. *LIMS*

See *Table 2* for LIMS characteristics.

2. *EHR*

The current EHR consists of:

- ICE desktop (ordering and receiving laboratory results, producing electronic discharge letters)
- EPMA (prescriptions, and provides point-of-prescribing allergy and drug-drug interaction alerts)
- Centricity PACS (imaging)

Requirements

- Genotyping will be carried out by the accredited Liverpool Clinical Laboratories service. The raw genotyped data will need storage in a, likely, new database
- De-identification of genotyped data will be required
- Raw data interpretation: we will utilize bio.logis and transfer the relevant anonymous data to bio.logis for interpretation and receipt of the individualized report.
- The individualized report need to undergo local quality control checks, and will need to be married up again to the correct patient electronically prior to release onto ICE desktop.
- If the MSC pocket card is used, the card should indicate that the patient participates in a clinical trial.
- A way to generate and print the MSC cards based on the interpreted PGx results (haplotypes, phenotypes) needs to be established.

Italy

Setting

In Italy, patients will be recruited from the Medical Oncology and Radiotherapy Oncology Unit of the National Cancer Institute in Aviano.

PGx implementation workflow

1. *Identifying patient for PGx testing eligibility*

2. Web- and paper-based reminders will be used to remind clinicians that a patient is eligible for inclusion into the study.*Ordering PGx test*

The request for PGx testing is usually sent electronically by the prescribing physician. For inpatients, blood samples are collected and sent directly to the lab. For outpatients, blood samples are sent via DHL and arrive at the lab within 24 hours.

3. *Conducting PGx test*

Currently, samples for genotyping are pooled every Monday. A patient ID is assigned to each sample. Results are usually delivered within 48-72 hours. For U-PGx, PGx testing will be performed using a standardized genotyping platform provided by bio.logis.

4. *Storage of raw PGx data*

Currently, de-identified raw PGx data are saved in an Excel database stored in a lab server with limited personnel access. In this database, raw PGx data is only associated to the patient's ID. For U-PGx a separate database for storing raw genetic data will be created.

5. *Interpretation of raw PGx data*

At the moment, the interpretation of raw PGx results is conducted manually.

6. *Storage of interpreted PGx data (=phenotypes)*

Currently, the results are manually entered into a centralized software that stores the data and generates a structured PDF file. The option to integrate interpreted PGx data in the hospital information system will be evaluated.

7. *Generating PGx report and transferring it to intended users*

- A printout of the PDF report is forwarded to the ordering physician who puts it in the patient's paper record.
- In the inpatient setting, results are also accessible as single text documents via the EHR. However, the accessibility of the patient's clinical records depends on the informed consent signed by the patient when registered at the first CRO hospital access.
- For U-PGx, it would also be possible to store a PDF in a centralized repository, separated from ordinary health documents that would be accessible to all physicians inside the region (north-east Italy). For patients outside the region, only paper reports are available.

IT Infrastructure

1. *LIMS*

See *Table 2* for LIMS characteristics.

2. EHR

An EHR that contains a part of the patient's medical history, including lab results, is available in the inpatient setting and partly also in the outpatient setting. These records are accessible to the physician as single text documents that can be selected from a list.

Requirements

- For U-PGx, at new database will be created for the storage of raw PGx data.
- The raw PGx results provided by the genotyping platform need to be fed into this database.
- De-identification is required before PGx data can be stored in the database.
- A way to automatically generate a PDF report that contains the interpreted PGx results and recommendations needs to be established.
- The PGx report should contain a reference to the clinical trial the patient is enrolled in.
- The generated PDF file needs to be transferred to the EHR.
- If a centralized repository that is accessible to physicians inside the region will be established, the PDF report needs to be transferred to this repository.
- A way to generate and print the MSC cards based on the interpreted PGx results (haplotypes, phenotypes) needs to be established.
- If the MSC pocket card is used, the card should contain a reference to the clinical trial the patient is enrolled in.
- Web- and paper-based reminders for identifying eligible patients are required.

Spain

Setting

Patients will be recruited at the departments of Pharmacy and Cardiology of the San Cecilio University Hospital in Granada, with a focus on patients with vascular disease, unstable angina and acute coronary syndrome undergoing percutaneous coronary intervention with stent.

PGx implementation workflow

1. Identifying patient for PGx testing eligibility

The preferable option for U-PGx would be to implement an alert system that reminds the physician at the stage of drug prescribing that the patient is eligible for PGx testing. An alternative option would be to screen the list of drug prescriptions at the end of the day.

2. Ordering PGx test

If the patient agrees to participate in the PGx project, the physician contacts the clinical pharmacist. The patient signs an informed consent form, and a saliva sample is collected and sent to the lab.

3. Conducting PGx test

It is anticipated that the PGx tests can be conducted within the lab of the pharmacy. For U-PGx, PGx testing will be performed using a standardized genotyping platform provided by bio.logis. Currently, samples are processed twice peer week and the results are available within 24 hours. For U-PGx samples can be processed daily.

4. Storage of raw PGx data

The raw results can be stored in the local LIMS.

5. Interpretation of raw PGx data

Currently, the interpretation of raw PGx data is done manually. For U-PGx, the software provided by bio.logis will be used.

6. Storage of interpreted PGx data (=phenotypes)

PGx reports are generated manually via the EHR and stored as HTML files. Reports can also be saved as PDF files.

7. Generating PGx report and transferring it to intended users

Currently, PGx text reports are generated via the EHR.

IT Infrastructure

1. LIMS

See *Table 2* for LIMS characteristics.

2. EHR

An EHR infrastructure is available both in the hospital and primary care setting. The EHR has the capability to generate active alerts which is currently used for drug allergy alerts.

Requirements

- Active alerts to remind the physician that a relevant PGx drug is prescribed should be implemented in the local EHR.
- The raw PGx results provided by the genotyping platform need to be fed into the LIMS database.
- A way to automatically generate a PDF report that contains the interpreted PGx results and recommendations needs to be established.
- The generated PDF file needs to be transferred to the EHR.
- The PGx report should indicate that the patient participates in a clinical trial, and that any modification of the treatment during the follow-up period should be communicated to the pharmacy service.
- A way to generate and print the MSC cards based on the interpreted PGx results (haplotypes, phenotypes) needs to be established.
- If the MSC pocket card is used, the card should indicate that the patient participates in a clinical trial, and that any modification of the treatment during the follow-up period should be communicated to the pharmacy service.

Austria

Setting

At the Vienna General Hospital, pre-emptive PGx testing will be implemented at the Division of Nephrology and Dialysis, focusing on patients who are scheduled for a kidney transplantation.

PGx implementation workflow

1. Identifying patient for PGx testing eligibility

Patients who are scheduled for a kidney transplantation and are anticipated to receive a relevant PGx medication will be eligible for pre-emptive PGx testing. Furthermore, prevalent ambulatory patients who receive a novel prescription of a PGx drug will be included into the study. Web- and

paper-based reminders will be used to remind clinicians that a patient is eligible for inclusion into the study.

2. *Ordering PGx test*

The PGx testing will preferably be performed in advance for patients who are listed for a kidney transplantation. This will, however, not be possible for the majority of patients due to various reasons (e.g. large geographical distance between patient residence and transplantation center). Thus, for the majority of transplant patients, the blood sample will be collected at the time of actual hospital admittance for transplantation.

3. *Conducting PGx test*

Currently, PGx tests are conducted once a week, and DNA isolation is conducted three times a week. DNA isolation takes only a couple of hours, whereas the processing of accumulated probes for PGx testing takes about 2 days. For U-PGx, PGx testing will be performed using a standardized genotyping platform provided by bio.logis.

4. *Storage of raw PGx data*

Raw PGx results are stored as CSV files in the LIMS.

5. *Interpretation of raw PGx data*

Currently, the interpretation of the raw PGx data and the preparation of the PGx report are conducted manually.

6. *Storage of interpreted PGx data (=phenotypes)*

PGx reports are stored in the LIMS.

7. *Generating PGx report and transferring it to intended users*

At the moment, printed PGx reports are delivered exclusively to the ordering physician.

IT Infrastructure

1. *LIMS*

See *Table 2* for LIMS characteristics.

- License fee for interfaces: 2000€
- Connection via VPN is possible but getting a permit probably not.

2. *EHR*

- AKIM/i.s.h med
- It may be possible to include the medications which are affected by the patient's genotype in the patient's file in AKIM.

Requirements

- If PGx data will be transferred to AKIM, a method for data anonymization and re-identification is required.
- A way to display the drug substances that are critically affected by the patient's genotype should be created.

- A way to generate and print the MSC cards based on the interpreted PGx results (haplotypes, phenotypes) may be established.
- Web- and paper-based reminders for identifying eligible patients are required.

Slovenia

Setting

In Slovenia, patients will be recruited from three different sites: (1) The Dialysis Center of the University Clinical Center Ljubljana, (2) the Rheumatology Department of the University Clinical Center Ljubljana and (3) a Community Health Care Center.

PGx implementation workflow

1. *Identifying patient for PGx testing eligibility*

Patients eligible for PGx testing will be identified based on (1) a novel prescription of a PGx drug, and (2) based on an upcoming transplantation. Thorough communication, as well as web- and paper-based reminder tools will ensure that all involved physicians are aware of the relevant PGx drugs and the demands of the project.

2. *Ordering PGx test*

A blood sample is collected and sent to the lab.

3. *Conducting PGx test*

PGx tests are conducted at the Phamacogenetics laboratory of the Faculty of Medicine at the University Clinical Center Ljubljana. For U-PGx, PGx testing will be performed using a standardized genotyping platform provided by bio.logis.

4. *Storage of raw PGx data*

Raw de-identified PGx data are stored in a database of the local lab IT system.

5. *Interpretation of raw PGx data*

Currently, the preparation of the PGx results reports, including the translation from SNPs to phenotypes and the matching of recommendations, is a manual step conducted by the laboratory head.

6. *Storage of interpreted PGx data (=phenotypes)*

A paper-based PGx report including relevant recommendations is created by the laboratory head and added to the patient's paper-based record.

7. *Generating PGx report and transferring it to intended users*

A paper-based PGx report including relevant recommendations is created by the laboratory head, added to the patient's paper-based record and delivered to the treating physician.

IT Infrastructure

1. *LIMS*

See *Table 2* for LIMS characteristics.

2. EHR

At the moment, no consistent EHR infrastructure is available at the Slovenian implementation sites, and there is no way to generate active alerts. However, the implementation of an EHR infrastructure with this capability will be driven forward for both in- and outpatient settings within the next years.

Requirements

- The raw PGx results provided by the genotyping platform need to be fed into the existing database, or a new way for storing the raw results needs to be established for U-PGx.
- A way to automatically generate paper-based PGx reports that contain the interpreted PGx results and DPWG recommendations needs to be established.
- A way to generate and print the MSC cards based on the interpreted PGx results (haplotypes, phenotypes) needs to be established.

Web- and paper-based reminders for identifying eligible patients are required.

Greece

Setting

The department of Pharmacy of the University of Patras has established a nation-wide network of psychiatrists in several cities in Greece. Also, the department has strong collaboration ties with the Hellenic Society of Medical Oncology, which gives access to various oncology clinics in Greece that would be incorporated in the project. Next to these specialties, there is expressed interest to implement pre-emptive pharmacogenomics in transplantation and cardiology clinics in Athens and Patras.

PGx implementation workflow

1. *Identifying patient for PGx testing eligibility*

   Patients eligible for PGx testing will be identified based on the first prescription of a relevant drug. Close personal contact on a daily basis and paper-based reminders will ensure that all involved physicians are aware of the relevant PGx drugs and the demands of the project. Patients will be also educated and hence, encouraged to share their PGx reports with treating physicians.

2. *Ordering PGx test*

   A blood/saliva sample is collected and sent to the department of Pharmacy lab for analysis.

3. *Conducting PGx test*

   For U-PGx, PGx testing will be performed using a standardized genotyping platform provided by bio.logis.

4. *Storage of raw PGx data*

   Raw PGx results are de-identified, assigned an in-house code and stored at the local laboratory as CSV files.

5. *Interpretation of raw PGx data*

   The translation from raw PGx data is currently conducted by a self-developed in-house tool (ePGA).

6. *Storage of interpreted PGx data (=phenotypes)*

   PGx reports are stored in the patient's paper-based record.

7. Generating PGx report and transferring it to intended users

Paper-based PGx reports are delivered to the patient and the ordering physician. Patients are encouraged to share their PGx report with all other treating physicians.

IT Infrastructure

1. LIMS

No LIMS infrastructure is available.

2. EHR

No EHR infrastructure is available.

Requirements

- The raw PGx results provided by the genotyping platform need to be fed into the existing repository, or a new way for storing the raw results needs to be established for U-PGx.
- De-identification is required before PGx data can be stored.
- A way to automatically generate paper-based PGx reports that contain the interpreted PGx results and DPWG recommendations needs to be established.
- A way to generate and print the MSC cards based on the interpreted PGx results (haplotypes, phenotypes) needs to be established.
- Web- and paper-based reminders for identifying eligible patients are required.

Summary

Table 1 summarizes the general infrastructure characteristics at the implementation sites, and *Table 2* provides information about the LIMS infrastructure. *Figure 2* provides an overview of the workflow for implementing pre-emptive PGx testing at each of the 7 implementation sites, broken down by basic steps from identifying patients to transferring PGx results to physicians and pharmacists. *Table 3* lists which CDS interventions will be deployed at the different implementation sites. A summary of the requirements of each implementation site can be found in *Table 4.*

Infrastructure characteristics	NL	UK	I	E	A	SLO	GR
EHR inpatient setting	Yes	Yes	Partially	Yes	Partially	Partially	No
Text reports	Yes	Yes	Yes	Yes	Yes	Yes	Yes
Active CDS	Yes	Yes (for allergies and DDI)	No	Yes (for allergies)	No	No	No
Passive CDS	No	Yes	Yes	Yes	Yes (?)	No	No
LIMS	Yes	Yes	Yes	Yes	Yes	Yes	No
Structured laboratory results	Yes	Yes	Yes	Yes	Yes	No	No
Storage of raw PGx results	Yes	Yes	Yes	Yes	Yes	Yes	Yes
EHR outpatient setting	Yes	Partially	Partially	Yes	No	No	No

Table 1: Overview of existing health IT infrastructure at the 7 different implementation sites

**LIMS characteristics**	**NL**	**UK**	**I**	**E**	**A**	**SLO**	**GR**
Vendor	MIPS	TBD	NoemaLife	Werfren / Francisco Soria Melguizo	SYSMEX	TBD	N/A
Product	GLIMS	TBD	Dnlab	Modulab / Microb	MOLIS	TBD	N/A
Contact	Elma Straathof (LUMC, E.A.T.Straathof@lumc.nl)	TBD	Insiel: Gilda De Marco (gilda.demarco@insiel.it)	At the moment Rosario Moreno Aguilar (rosario.moreno.aguilar.sspa@juntadeandalucia.es)	TBD	TBD	N/A
HL7 or LDT support or other interfaces/APIs? Versions?	HL7 for result reporting ASTM interfacing IN GLIMS	TBD	Interface HL7, clinical reports: XML – CDA level 2	HL7, V2.5	Yes	TBD	N/A
Internal text format and encoding? What kind of report engine is used, e.g. Word? Integration of HTML-content possible?	Reporting is possible in our Hospital Information System (HIX, vendor: ChipSoft, contact: LUMC)	TBD	Report format: PDF	PDF or XSL templates and XML report	Yes	TBD	N/A
Possibility to connect to external services through HTTPS (using client certificates) or VPN?	No	TBD	Yes, VPN	VPN	Yes, VPN	TBD	N/A
Customization possible without vendor?	Yes, only after approval of LAB-ICT LUMC	TBD	No	No	Yes	TBD	N/A

Table 2 Overview of LIMS characteristics at the 7 different implementation sites

Planned CDS intervention	NL	UK	I	E	A	SLO	GR
Active alerts via EHR	xx						
Paper-based PGx reports			x		xx	xx	xx
Digital PGx reports via EHR		xx	xx	xx	x		
MSC system	x	x	x	x	x	x	x

Table 3: Planned CDS interventions at each implementation site.

Each clinical site will use one of the listed CDS intervention as their main method for providing physicians and pharmacists with patient-specific drug dosing recommendations, depending on their existing infrastructure and IT capabilities. Furthermore, each implementation site will deploy additional methods to facilitate the transfer of results between different health care settings (e.g. inpatient and outpatient setting). Main methods are marked with xx, adjunct methods are marked with x.

Requirements	NL	UK	I	E	A	SLO	GR
Implementation of active reminder alerts for identifying eligible patients	x			(x)			
Web- and paper-based reminders for identifying eligible patients		x	x		x	x	x
Transfer of raw PGx results from genotyping platform to database/repository	x	x	x	x	x	x	x
De-identification of raw PGx data before storing		x	x		x		x
Automatic generation of PGx report with results and DPWG recommendations		x	x	x	x	x	x
Transfer of PGx report file to EHR		x	x	x			
Transfer of structured, interpreted PGx results to EHR for active CDS	x						
Customized PGx recommendations (apart from translation)							
Generate and print MSC cards based on interpreted PGx results (haplotypes, phenotypes)	x	x	x	x	x	x	x
Customization of PGx report/MSC card		x	x	x			

Table 4 Overview of requirements of the 7 different implementation sites

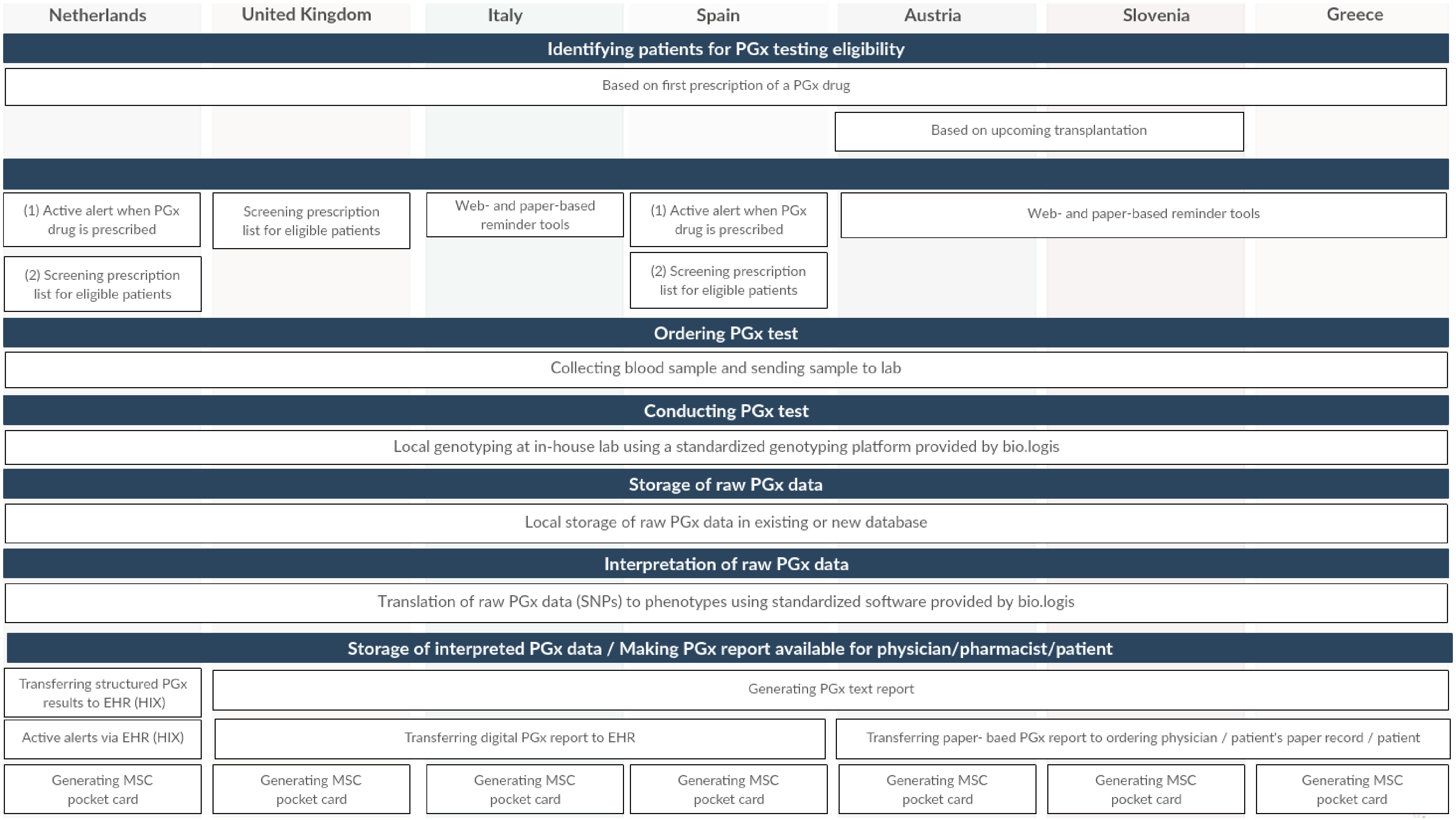

Figure 2: Anticipated workflow for PGx testing and delivery of results at the 7 implementation

Abbreviations

**CDS**	Clinical Decision Support
**EHR**	Electronic Health Record
**EPMA**	Electronic Prescribing and Medicine Administration
**ICT**	Information and communications technology
**LIMS**	Laboratory Information Management System
**LUMC**	Leiden University Medical Center
**MSC**	Medication Safety Code
**PGx**	Pharmacogenomics

List of tables

Table 1: Overview of existing health IT infrastructure at the 7 different implementation sites 17
Table 2 Overview of LIMS characteristics at the 7 different implementation sites 18
Table 3: Planned CDS interventions at each implementation site. 19
Table 4 Overview of requirements of the 7 different implementation sites 19

List of figures

Figure 1: Illustration of the basic workflow for U-PGx. 6
Figure 2: Anticipated workflow for PGx testing and delivery of results at the 7 implementation 20

References

1. Hoffman JM, Haidar CE, Wilkinson MR, Crews KR, Baker DK, Kornegay NM, et al. PG4KDS: a model for the clinical implementation of pre-emptive pharmacogenetics. Am J Med Genet C Semin Med Genet. 2014 Mar;166C(1):45–55.

2. Pulley JM, Denny JC, Peterson JF, Bernard GR, Vnencak-Jones CL, Ramirez AH, et al. Operational implementation of prospective genotyping for personalized medicine: the design of the Vanderbilt PREDICT project. Clin Pharmacol Ther. 2012 Jul;92(1):87–95.

3. Gottesman O, Scott SA, Ellis SB, Overby CL, Ludtke A, Hulot J-S, et al. The CLIPMERGE PGx Program: clinical implementation of personalized medicine through electronic health records and genomics-pharmacogenomics. Clin Pharmacol Ther. 2013 Aug;94(2):214–7.

4. Johnson JA, Elsey AR, Clare-Salzler MJ, Nessl D, Conlon M, Nelson DR. Institutional profile: University of Florida and Shands Hospital Personalized Medicine Program: clinical implementation of pharmacogenetics. Pharmacogenomics. 2013 May;14(7):723–6.

5. Johnson JA, Burkley BM, Langaee TY, Clare-Salzler MJ, Klein TE, Altman RB. Implementing personalized medicine: development of a cost-effective customized pharmacogenetics genotyping array. Clin Pharmacol Ther. 2012 Oct;92(4):437–9.

6. O'Donnell PH, Bush A, Spitz J, Danahey K, Saner D, Das S, et al. The 1200 patients project: creating a new medical model system for clinical implementation of pharmacogenomics. Clin Pharmacol Ther. 2012 Oct;92(4):446–9.

7. O'Donnell PH, Danahey K, Jacobs M, Wadhwa NR, Yuen S, Bush A, et al. Adoption of a clinical pharmacogenomics implementation program during outpatient care--initial results of the University of Chicago "1,200 Patients Project." Am J Med Genet C Semin Med Genet. 2014 Mar;166C(1):68–75.

8. Lunenburg CA, van Staveren MC, Gelderblom H, Guchelaar H-J, Swen JJ. Evaluation of clinical implementation of prospective DPYD genotyping in 5-fluorouracil- or capecitabine-treated patients. Pharmacogenomics. 2016 May;17(7):721–9.

9. Ten Brink MH, van der Straaten T, Bouwsma H, Baak-Pablo R, Guchelaar HJ, Swen JJ. Pharmacogenetics in transplant patients: mind the mix. Clin Pharmacol Ther. 2013 Oct;94(4):443–4.
