# Supplementary material for: Pharmacogenomics decision support in the U-PGx project: Results and advice from clinical implementation across seven European countries
Source: PLoS One. 2022 Jun 8;17(6):e0268534. doi: 10.1371/journal.pone.0268534 (PMC9176797; doi:10.1371/journal.pone.0268534)
Supplement: S2 File — Analysis of the U-PGx CDS infrastructure in relation to the European regulatory and legal landscape. (PDF) [file pone.0268534.s002.pdf]

Analysis of European regulatory requirements

Contents

Introduction 2
European medical device regulation and relevant classification schemes 2
European Medical Devices Directive (MDD) 93/42/EEC 2
Software classification schemes 3
MEDDEV 2.1/6 3
Manual on Borderline and Classification 6
UK: Medicines and Healthcare Products Regulatory Agency 6
IMDRF (International Medical Device Regulator Forums) 7
Medical Device Approval & Certification 7
Interpretation & consequences for U-PGx 7
GIMS 7
MSC system 8
Conclusions 9
Abbreviations 9
References 9

Introduction

The successful implementation of pharmacogenomic (PGx) testing in the context of U-PGx will largely depend on the provision of suitable information and communication technologies (ICT), including clinical decision support (CDS) systems. The following technologies will be used to ensure that the patients' PGx test results are available to clinicians and pharmacists at the point of care during the clinical trial phase of U-PGx:

- A **Genetic Information Management System (GIMS)** developed by the German company bio.logis: After genetic data is transferred from the local laboratory information management system (LIMS) to the GIMS using standard interfaces such as HL7, raw data is interpreted by the GIMS' genetic rule engine. For U-PGx sites with the capability of providing active CDS (only the Netherlands), PGx results will be transferred to the local electronic health record (EHR) in a structured format. For all other clinical sites, a digital PGx report will be generated by the GIMS which can then be transferred to the local EHR for passive CDS, or can be printed and filed in the patient's paper-based record.

- The **Medication Safety Code (MSC) system**: The MSC system is a mobile-based CDS system developed at the Medical University of Vienna that enables the quick retrieval of patient-relevant PGx drug dosing guidelines. The main component of the MSC system is a QR code that stores the patient's encoded PGx results. It can be decoded and interpreted by common smartphones and other devices. After scanning the QR code, the medical professional is led to a website that provides drug dosing recommendations customized to the PGx profile of the patient. The MSC can be included in paper-based lab reports, or it can be printed on personalized pocket cards. Patients can carry these cards in their wallets and display them to medical professionals when pharmacotherapy is initiated or altered. In the context of U-PGx, the MSC system is aimed to serve as an auxiliary tool to maximize the accessibility and sharing of PGx results within and between different health care settings and health care professionals.

The development of ICTs for deployment in clinical care makes conformity with legal and regulatory requirements, such as the European medical device regulation, indispensable. The aim of this deliverable is therefore, to analyse these requirements with regard to the ICTs utilized in U-PGx. The following questions will be considered:

- Which directives and classification schemes are relevant for the ICTs used in U-PGx?
- Do the U-PGx ICTs qualify as medical devices according to these regulations?
- Do the U-PGx ICTs need certification?

European medical device regulation and relevant classification schemes

European Medical Devices Directive (MDD) 93/42/EEC

According to the European Medical Devices Directive (MDD) 93/42/EEC, medical devices are defined as follows (1) :

“'medical device' means any instrument, apparatus, appliance, material or other article, whether used alone or in combination, including the software necessary for its proper application intended by the manufacturer to be used for human beings for the purpose of:

- *diagnosis, prevention, monitoring, treatment or alleviation of disease,*
- *diagnosis, monitoring, treatment, alleviation of or compensation for an injury or handicap,*
- *investigation, replacement or modification of the anatomy or of a physiological process,*
- *control of conception,*

and which does not achieve its principal intended action in or on the human body by pharmacological, immunological or metabolic means, but which may be assisted in its function by such means”

The purpose of the medical software refers to the manufacturer’s intended purpose which can be derived from the promotional materials for the device, such as brochures or the product’s website.

Software classification schemes

In addition to the European Medical Device Directive, several guidelines and classification schemes have been published by various organizations with the aim of assisting stakeholders in the decision if a software qualifies as a medical device.

MEDDEV 2.1/6

The MEDDEV guidelines have been published by the European Commission with the aim of facilitating the classification of medical devices. (2) MEDDEV 2.1/6 offers guidance on the decision if a standalone software product qualifies as a medical device. The document proposes six steps for deciding if software needs CE certification and provides a decision tree (see Figure 1):

*“**Decision step 1**: **If the stand alone software is a computer program, then it may be a medical device**. If the software is not a computer program, then it is a digital document and therefore not a medical device. Examples of computer programs are software applications, macros, scripts, dynamically linked libraries, batch files, style sheets and any document containing active formatting or filtering instructions. Examples of digital documents are image files, DICOM files, digital ECG recordings, numerical results from tests and electronic health records (EHR).*
***Decision step 2**: if the software is incorporated into a medical device rather than standalone software, it must be considered as part of that medical device in the regulatory process of that device. If it is standalone software, proceed to decision step 3.*
***Decision step 3: if the software does not perform an action on data, or performs an action limited to storage, archival, communication, ‘simple search’ or lossless compression (i.e. using a compression procedure that allows the exact reconstruction of the original data) it is not a medical device.** Altering the representation of data for embellishment purposes does not make the software a medical device. In other cases, including where the software alters the representation of data for a medical purpose, it could be a medical device.*

> ***'Simple search' refers to the retrieval of records by matching record metadata against record search criteria, e.g. library functions. Simple search does not include software which provides interpretative search results, e.g. to identify medical findings in health records or on medical images.*** *Software which is intended to create or modify medical information might be qualified as a medical device. If such alterations are made to facilitate the perceptual and/or interpretative tasks performed by the healthcare professionals when reviewing medical information, (e.g. when searching the image for findings that support a clinical hypothesis as to the diagnosis or evolution of therapy) the software could be a medical device.*
>
> ***Decision step 4:*** *an example of software for the benefit of individual patients is software intended to be used for the evaluation of patient data to support or influence the medical care provided to that patient.* ***Examples of software which are not considered as being for the benefit of individual patients are those which aggregate population data, provide generic diagnostic or treatment pathways, scientific literature, medical atlases, models and templates as well as software for epidemiologic studies or registers.***
>
> ***Decision step 5: if the manufacturer specifically intends the software to be used for any of the purposes listed in Article 1 of Directive 93/42/EEC, then the software shall be qualified as a medical device****. However, if only a non-medical purpose is intended by the manufacturer, such as invoicing or staff planning, it is not a medical device.*
>
> ***Decision step 6:*** *if the software is an accessory to a medical device, it is not a medical device, but it falls under Directive 93/42/EEC. The legal definition of 'putting into service' requires that a device is made available to the final user/operator as being ready for use on the Community market. Software made available to the user over the internet (directly or via download) or via in vitro diagnostic commercial services, which is qualified as a medical device, is subject to the medical devices directives."*

Furthermore, the MEDDEV guidelines explicitly state that medical decision support software is generally classified as a medical device:

> ***"****In general, they are computer based tools which combine medical knowledge databases and algorithms with patient specific data. They are intended to provide healthcare professionals and/or users with recommendations for diagnosis, prognosis, monitoring and treatment of individual patients. Based on steps 3, 4, and 5 of Figure 1, they are qualified as medical devices."*

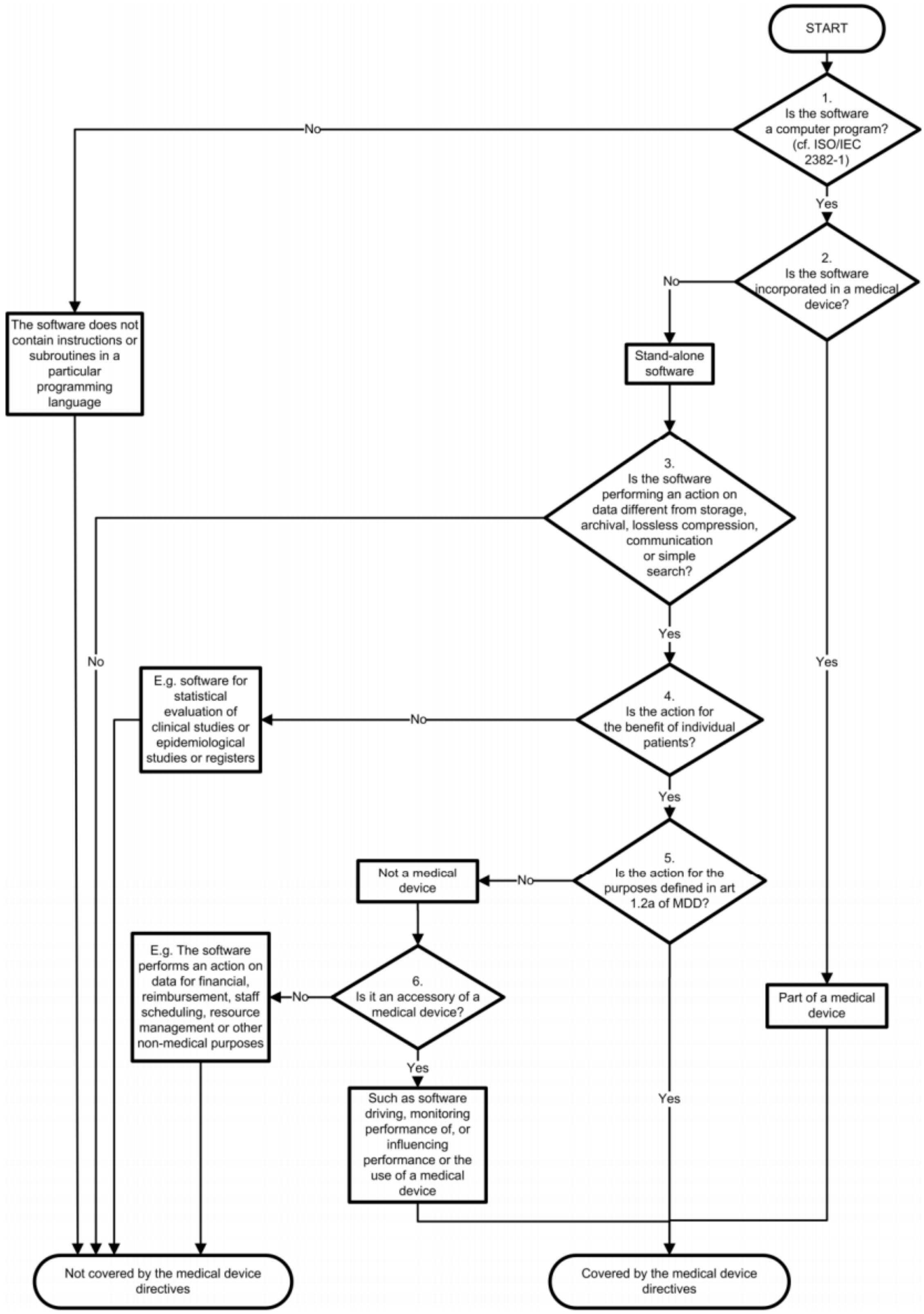

Figure 1: MEDDEV 2.1/6 Decision diagram for standalone software

COCIR (European Coordination Committee of the Radiological, Electromedical and Healthcare It Industry)

The European Coordination Committee of the Radiological, Electromedical and Healthcare IT Industry has published a document that recites the decision steps listed in the MEDDEV guidelines (see above) and additionally contains a similar decision diagram for qualification of software as medical device. (3)

Manual on Borderline and Classification

The European Commission's Manual on Borderline and Classification provides guidance on the classification of medical devices based on exemplary cases, including a section on software and mobile applications. (4) The document lists several examples of software applications and discusses if they should be qualified as medical devices, e.g.:

- **A mobile application for processing ECGs**: According to the document, this application should be qualified as a medical device because it "*uses signal data from an external source and processes it to an ECG waveform thereby performing an action on data other than just storage, for the medical benefit of individual patients.*"
- **A mobile application for the communication between patient and caregivers while giving birth:** "*This app, which is not incorporated in a medical device, performs an action on data limited to storage and simple search. In accordance with the guidance document MEDDEV 2.1/6 rev.1, a mobile app for the communication between patients and caregivers while giving birth should not be qualified as a medical device."*
- **A mobile medical application for viewing the anatomy of the human body:** "*Although the app is not only performing simple data search it is not used directly for the medical benefit of the individual patients. In light of this, this app should not be qualified as a medical device."*
- **An application which allows for faster consulting/reading of an international guideline regarding the Classification of Malignant Tumours (TNM): "***This application should not be qualified as a medical device because "The software does not perform an action on data other than simple search function, as per in MEDDEV Guidance 2.1/6."*

UK: Medicines and Healthcare Products Regulatory Agency

The UK Medicines and Healthcare products Regulatory Agency has published a document that offers guidance on which software applications are likely to qualify as medical devices and how to comply with the legal requirements. (5) This document describes medical decision support software as follows:

> *"Decision support software is usually considered a medical device when it applies automated reasoning such as a simple calculation, an algorithm or a more complex series of calculations. For example, dose calculations, symptom tracking, clinicians guides to help when making decisions in healthcare. This is likely to fall within the scope of the medical devices directives.*
> *Some decision support software may not be considered to be a medical device if it exists only to provide information to enable a healthcare professional to make a clinical decision, as they ultimately rely on their own knowledge. However, if the software/ app performs a calculation or interprets or interpolates data and the healthcare professional does not review the raw data, then this software may be*

considered a medical device. Apps are increasingly being used by clinicians who will rely on the outputs from this software and may not review the source/raw data."

IMDRF (International Medical Device Regulator Forums)

The International Medical Device Regulators Forum (IMDRF), a working group consisting of global medical device regulators, has published a document to guide the decision whether a software application qualifies as a medical device. (6)

Medical Device Approval & Certification

The classification of medical devices is regulated in Article IX of the Council Directive 93/42/EEC. (1) According to this directive, medical devices are divided into four groups, depending on their functionality (invasiveness) and risk assignment: Class I (non-invasive devices, low risk), IIa, IIb or III (high risk).

According to Article XVII of this directive, all medical devices must bear the CE marking of conformity when they are placed on the market unless the device is custom-made or intended for clinical investigation.

Interpretation & consequences for U-PGx

According to the European MDD and other relevant sources described above, software, including decision support systems, that interprets an individual patient's genetic data in general qualifies as a medical device, and therefore has to be certified. However, and as can be read in some of the sources described above, there are some exceptions to this, depending on the intended use and functionality of the medical software / application. In general, it can be concluded that standalone software that performs an action on data (e.g. calculations, interpretation algorithms) other than a simple search, that generates new specific information, or that performs an action on data for the benefit of an individual patient has to be certified as a medical device.

Since the bio.logis GIMS Diagnostic Report Module and the MSC system do not generate any new information, but instead apply a simple procedure to match existing information (i.e. guideline recommendations) to all patients with a certain genetic variant, they do not qualify as medical devices.

A closer analysis of the aspects that make it highly unlikely that the bio.logis GIMS Diagnostic Report Module and the MSC system would be considered medical devices is provided below.

GIMS

Two main facts support the assumption that the bio.logis GIMS Diagnostic Report Module that will be deployed for generating the genetic test reports at all U-PGx implementation sites does not qualify as a medical device:

1. According to MEDDEV 2.1/6, expert function software that generates new specific information would qualify as a medical device. However, GIMS does not generate any new data, but instead compiles case-related existing information.
2. The results of this matching and compiling process performed by the GIMS Diagnostic Report Module are valid for all patients with certain genetic variants rather than only for an individual patient.

Nevertheless, it was voluntarily decided by bio.logis to certify their Diagnostic Report module as a medical device.

ithin the first year of U-PGx, the bio.logis GIMS Diagnostic Report Module will be certified as a class I medical device according to the European Medical Devices Directive (MDD) 93/42/EEC and Medical Device Directive 2007/47 EC.

The certification is conducted in a two-stage process. In the first stage, the Diagnostic Report Module receives the seal "Qualitätsprodukt Internetmedizin" ("Quality product internet medicine"). The requirement catalogue for this seal is based on the European Medical Devices Directive's (MDD) requirements for the class I medical devices and the U.S. Food and Drug Administration's (FDA) requirements for class I medical devices. In the second stage, the GIMS Diagnostic Report Module will receive the CE certificate for medical devices.

The seal is awarded by the German Bundesverband Internetmedizin ("Federal Association Internet Medicine", http://www.bundesverbandinternetmedizin.de). For the certification process, bio.logis will be supported by the German company mpP Group (http://www.mpp-group.de).

The certification process is expected to be finished in August / September 2016.

MSC system

In consideration of the European MDD and the available classification schemes described in the Results section, it can be reasonably assumed that the MSC system does not qualify as a medical device, and therefore does not need certification.

There are a number of factors that support this assumption:

1. The MSC system's core functionality is to speed up the process of looking up recommendations manually. It makes it easier for the user to access up-to-date clinical guidelines.
2. The MSC system does not perform any calculations, nor does it interpret raw genetic data by using complex algorithms. Its functionality is reduced to a simple search, i.e. matching phenotypes with relevant recommendations.
3. The interpretation of raw genotyping data into phenotypes is not performed by the MSC system Instead, the MSC system uses already interpreted data (i.e. phenotypes) and matches them with clinical guidelines.

4. The data (i.e. phenotypes) used by the MSC system to search for the matching guideline and recommendation are accessible to the user by a quick glance at the pocket card. This transparency enables the user to search for the matching recommendations manually at any time, if needed.

Conclusions

According to the relevant European directives and regulations, the bio.logis GIMS that will be used to generate the PGx reports for U-PGx (bio.logis GIMS) does not qualify as a medical device, but will be certified on a voluntary basis. It will be certified as a class I medical device according to the European Medical Devices Directive (MDD) 93/42/EEC and Medical Device Directive 2007/47 EC. The certification process is expected to be finished in August / September 2016.

With regard to the European MDD and available guidance documents, the MSC system does not qualify as a medical device, and therefore does not require certification.

Abbreviations

CDS	Clinical Decision Support
EHR	Electronic Health Record
GIMS	Genetic Information Management System
LIMS	Laboratory Information Management System
MDD	Medical Devices Directive
MSC	Medication Safety Code
PGx	Pharmacogenomics

References

1. Council of the European Union. Council Directive concerning medical devices 93/42/EEC [Internet]. Jul 12, 1993. Available from: http://eur-lex.europa.eu/legal-content/EN/TXT/?uri=CELEX%3A31993L0042

2. European Commission. MEDDEV 2.1/6: Guidelines on the qualification and classification of stand alone software used in healthcare within the regulatory framework of medical devices [Internet]. 2012 [cited 2016 May 3]. Available from: http://ec.europa.eu/growth/sectors/medical-devices/guidance/index_en.htm

3. COCIR (European Coordination Committee of the Radiological, Electromedical and Healthcare It Industry). COCIR Contribution: Decision diagram for qualification of software as medical device [Internet]. 2010 [cited 2016 May 4]. Available from: http://www.cocir.org/fileadmin/Position_Paper_2010/cocir_medical_software_qualification_as_medical_device_-_22_nov_2010.pdf

4. European Commission. Manual on borderline and classification in the community: regulatory framework for medical devices [Internet]. [cited 2016 May 3]. Available from: http://ec.europa.eu/DocsRoom/documents/12867/attachments/1/translations/en/renditions/native

5. Medical device stand-alone software including apps - GOV.UK [Internet]. [cited 2016 Apr 13]. Available from: https://www.gov.uk/government/publications/medical-devices-software-applications-apps/medical-device-stand-alone-software-including-apps#decision-support-or-decision-making-software

6. IMDRF Software as a Medical Device (SaMD) Working Group. Software as a Medical Device: Possible Framework for Risk Categorization and Corresponding Considerations [Internet]. 2014 [cited 2016 May 4]. Available from: http://www.imdrf.org/docs/imdrf/final/technical/imdrf-tech-140918-samd-framework-risk-categorization-141013.pdf
