# Supplementary material for: Pharmacogenomics decision support in the U-PGx project: Results and advice from clinical implementation across seven European countries
Source: PLoS One. 2022 Jun 8;17(6):e0268534. doi: 10.1371/journal.pone.0268534 (PMC9176797; doi:10.1371/journal.pone.0268534)
Supplement: S3 File — Questionnaire to identify potential risks and problems of the implementation and results. (PDF) [file pone.0268534.s003.pdf]

U-PGx Risk Assessment Survey

(untitled)

ID 54

Dear U-PGx participant,

The aim of this survey is to **identify potential risks and problems** that may hinder the timely provision and implementation of the genotyping and IT technologies that are indispensable for the success of the clinical trial.

On the following pages you will be asked to list any risks or potential problems you are expecting to encounter (or may already encountered), and to rate these problems based on their estimated probability and impact. **Please be critical and list all potential risks and problems that come to your mind**—we will use this information to devise mitigation strategies to keep the project on track.

- The survey will take no more than a **few minutes**.
- You can also **pause** the survey and **continue it at a later time,** if needed; the results are saved automatically.
- The survey can also be completed by more than 1 person per institution (e.g. clinical staff + IT staff).
- You can complete the survey a second time if you need more than the 5 default text fields per problem category.

Please complete the survey by no later than June 6th.

Thank you!

(untitled)

5

1. Please select your institution: *

- LUMC (Leiden University Medical Center)
- PHUL (Royal Liverpool University Hospital)
- SASG (San Cecilio University Hospital Granada)
- MUWV (Medical University of Vienna)
- UPAT (University of Patras)
- ULMF (University of Ljubljana)
- CROA (Centro di Riferimento Oncologico, Aviano)
- Bio.logis
- KNMP

55

To help you think about potential issues at your institution, we have compiled this **roadmap** that roughly summarizes what lies ahead of us in the next months, and gives examples of potential problems that could emerge:

- Each implementation site needs:
  - an **IT contact person** who will be responsible for managing the implementation of ICT tools into the existing infrastructure.
  - a **lab contact person** who will be responsible for managing the implementation of the genotyping technologies.
  - *Examples of potential problems: difficulties in finding staff? enough time resources? reassignment of staff to be expected? Communication problems?*
- The **genotyping platform** needs to be **selected, ordered and provided** to the implementation sites. **Staff** needs to be **trained**.
  - *Examples of potential problems: enough staff for processing of samples? Timely provision of genotyping platform? Enough time for training of staff? Communication problems?*
- The **bio.logis system** that generates the **PGx reports** needs to be **finalized** (knowledgebase, report structure) and **implemented** at each clinical site.
  - *Examples of potential problems: enough and appropriate staff? Timely finalization? Enough time scheduled for integration with existing infrastructure (if applicable)? Communication problems?*
- The **healthcare professionals** who will order the PGx tests / use the PGx reports need to be **trained**.
  - *Examples of potential problems: recruitment of physicians / pharmacists? Enough time for training?*
- **Clinical trial**: involved health care professionals and patients need to adhere to the PGx recommendations
  - *Examples of potential problems: lack of motivation, poor adherence to PGx recommendations (e.g. across different hospital departments)*

LOGIC Show/hide trigger exists.

ID 6

2. Have you encountered or are you expecting to encounter any of the following problems? **Please select all that apply:**

- ☐ **Staffing problems** (e.g. difficulty getting sufficient or appropriate staff assigned to project, reassignment of staff)
- ☐ **Team coordination and communication problems** (e.g. lack of project management experience, need to integrate with parallel projects)
- ☐ **IT** / **Lab infrastructure problems** (e.g. change of EHR software)
- ☐ **Other changes at your implementation site** (e.g. moving into new building)
- ☐ **Other problems**

(untitled)

LOGIC Hidden unless: #2 Question "Have you encountered or are you expecting to encounter any of the following problems? **Please select all that apply:**" is one of the following answers ("**Staffing problems** (e.g. difficulty getting sufficient or appropriate staff assigned to project, reassignment of staff)")

ID 33

3. Please specify the **staffing problems** (e.g. difficulty getting sufficient or appropriate staff assigned to project, reassignment of staff). Please rate the expected likelihood and impact oft the problem(s).

	Probability	Impact	Problem description
Problem	Low (<20%)<br>Medium (20-80%)<br>High (>80%)	Minor<br>Medium<br>Severe	
Problem 2 (optional)	Low (<20%)<br>Medium (20-80%)<br>High (>80%)	Minor<br>Medium<br>Severe	
Problem 3 (optional)	Low (<20%)<br>Medium (20-80%)<br>High (>80%)	Minor<br>Medium<br>Severe	
Problem 4 (optional)	Low (<20%)<br>Medium (20-80%)<br>High (>80%)	Minor<br>Medium<br>Severe	
Problem 5 (optional)	Low (<20%)<br>Medium (20-80%)<br>High (>80%)	Minor<br>Medium<br>Severe	

Comments

(untitled)

LOGIC Hidden unless: #2 Question "Have you encountered or are you expecting to encounter any of the following problems? **Please select all that apply:**" is one of the following answers ("**Team coordination and communication problems** (e.g. lack of project management experience, need to integrate with parallel projects)")

ID 38

4. Please specify the **team coordination and communication problems** (e.g. lack of project management experience, need to integrate with parallel projects). Please rate the expected likelihood and impact oft the problem(s).

	Probability	Impact	Problem description
Problem	Low (<20%)<br>Medium (20-80%)<br>High (>80%)	Minor<br>Medium<br>Severe	
Problem 2 (optional)	Low (<20%)<br>Medium (20-80%)<br>High (>80%)	Minor<br>Medium<br>Severe	
Problem 3 (optional)	Low (<20%)<br>Medium (20-80%)<br>High (>80%)	Minor<br>Medium<br>Severe	
Problem 4 (optional)	Low (<20%)<br>Medium (20-80%)<br>High (>80%)	Minor<br>Medium<br>Severe	
Problem 5 (optional)	Low (<20%)<br>Medium (20-80%)<br>High (>80%)	Minor<br>Medium<br>Severe	

Comments

(untitled)

LOGIC Hidden unless: #2 Question "Have you encountered or are you expecting to encounter any of the following problems? **Please select all that apply:**" is one of the following answers ("**IT / Lab infrastructure problems** (e.g. change of EHR software)")

ID 42

5. Please specify the **IT / Lab infrastructure problems** (e.g. change of EHR software). Please rate the expected likelihood and impact oft the problem(s).

	Probability	Impact	Problem description
Problem	Low (<20%)<br>Medium (20-80%)<br>High (>80%)	Minor<br>Medium<br>Severe	
Problem 2 (optional)	Low (<20%)<br>Medium (20-80%)<br>High (>80%)	Minor<br>Medium<br>Severe	
Problem 3 (optional)	Low (<20%)<br>Medium (20-80%)<br>High (>80%)	Minor<br>Medium<br>Severe	
Problem 4 (optional)	Low (<20%)<br>Medium (20-80%)<br>High (>80%)	Minor<br>Medium<br>Severe	
Problem 5 (optional)	Low (<20%)<br>Medium (20-80%)<br>High (>80%)	Minor<br>Medium<br>Severe	

Comments

(untitled)

LOGIC Hidden unless: #2 Question "Have you encountered or are you expecting to encounter any of the following problems? **Please select all that apply:**" is one of the following answers ("**Other changes at your implementation site** (e.g. moving into new building)")

ID 46

6. Please specify the **other changes at your implementation site** (e.g. moving into new building). Please rate the expected likelihood and impact oft the problem(s).

	Probability	Impact	Problem description
Problem	Low (<20%)<br>Medium (20-80%)<br>High (>80%)	Minor<br>Medium<br>Severe	
Problem 2 (optional)	Low (<20%)<br>Medium (20-80%)<br>High (>80%)	Minor<br>Medium<br>Severe	
Problem 3 (optional)	Low (<20%)<br>Medium (20-80%)<br>High (>80%)	Minor<br>Medium<br>Severe	
Problem 4 (optional)	Low (<20%)<br>Medium (20-80%)<br>High (>80%)	Minor<br>Medium<br>Severe	
Problem 5 (optional)	Low (<20%)<br>Medium (20-80%)<br>High (>80%)	Minor<br>Medium<br>Severe	

Comments

(untitled)

LOGIC Hidden unless: #2 Question "Have you encountered or are you expecting to encounter any of the following problems? **Please select all that apply:**" is one of the following answers ("**Other problems**")

ID 50

7. Please specify the **other problems**. Please rate the expected likelihood and impact oft the problem(s).

	Probability	Impact	Problem description
Problem	Low (<20%)<br>Medium (20-80%)<br>High (>80%)	Minor<br>Medium<br>Severe	
Problem 2 (optional)	Low (<20%)<br>Medium (20-80%)<br>High (>80%)	Minor<br>Medium<br>Severe	
Problem 3 (optional)	Low (<20%)<br>Medium (20-80%)<br>High (>80%)	Minor<br>Medium<br>Severe	
Problem 4 (optional)	Low (<20%)<br>Medium (20-80%)<br>High (>80%)	Minor<br>Medium<br>Severe	
Problem 5 (optional)	Low (<20%)<br>Medium (20-80%)<br>High (>80%)	Minor<br>Medium<br>Severe	

Comments

(untitled)

ID 13

8. Do you have any other comments? (optional)

Thank You!

ID 1

Thank you!

Confirmation Email

To: Kathrin Blagec (kathrin.blagec@meduniwien.ac.at)

From: SurveyGizmo (notifications@surveygizmo.com)

Subject: New Response Notification

Risk assessment results

		Avoid<br>Control<br>Transfer	Avoid<br>Control<br>Transfer	Watch<br>Assume			
**Risk**	**Category**	**Institution**	**Probability**	**Impact**	**Severity**	**Mitigation strategies**	**Status**
problems for hiring personnel in staff due to bureaucratic problems: Comment: This issue has already been raised with the Project Coordinator	Staffing	CROA	Medium	Medium			Disabled
We are experiencing in personnel recruitement due to regulatory issues by the European Community. We are working on a solution. This should not affect the project activities	Staffing	CROA	High	Medium			Watch
I) time delays in setting up legal contract between Royal Liverpool hospital and Liverpool University have delayed our recruitment of a pharmacist to the study. Fortunately this is now occurring. Given that we have not received the study protocol yet and ideally the pharmacist will be responsible for the operational side of setting up the study, these factors combine to put pressure on ensuring the study starts on 1st January 2017.	Staffing	PHUL	Low	Medium			

We have experienced communication difficulties in getting ICT support staff 'on board' with the project. To resolve this, we are meeting in July with the regional director of ICT (at a few regional hospitals), who is known to have an interest in genomics. Hopefully, he will be willing to instruct some of his staff to help us in the U-PGx project.	Staffing	PHUL	Low	Medium			Watch
The move between hospital buildings will definitely occur during the U-PGx project, most likely in 2017. These building are on the same hospital campus though. Any impact on delivery of the project should be minimal because we are carrying out the standard-of-care 18 month arm first.	Changes at implementation site	PHUL	High	Minor			Assume
Interoperability Central Office manages all integration projects throughout the region, so their dedication to this project is not exclusive	Team coordination and communication	SASG	High	Medium			
It should be tested the connectivity between our Informatic Central Services (located in Sevilla) and all the external IT services (bio.logis) to the health network	Team coordination and communication	SASG	High	Medium			
**Collaboration and availability of different suppliers participating in the project**	Team coordination and	SASG	Medium	Severe			

	communication						
Financing for providers to adapt the computer systems to structured data, to manage messaging to reach them, moreover they must store the messages and generate alerts	Team coordination and communication	SASG	High	Medium			
The laboratory where we are going to get the PGx results is placed in another building and it doesn´t belong to our institution (it´s also a public laboratory). But fortunatelly, we are our own bosses in this research building.	IT / Lab infrastructure	SASG	High	Minor			
It´s necessary to know how we have to connect the crude data provided by the PGx platform with the LIMS in our hospital and how is the format of the register results	IT / Lab infrastructure	SASG	Medium	Medium		**Send out a fact sheet on the technical aspects to all IT contacts as soon as possible (new issues will probably arise once this is done)**	Control
**Informatic Central Services (located in Sevilla) must approve that the PGx report is incorporated into the EHR**	IT / Lab infrastructure	SASG	High	Severe			
Informatic Central Services requires that the file (for example: PGx report) which is incorporated into the EHR must be an XSL template and a message with the report in XML (pdf file is not permitted). This is new in our institution and we have no prior experience on it	IT / Lab infrastructure	SASG	High	Severe			

This year we are going to move to a new building. Similarly, the prescribing program is going to change. But hopefully, everything will be complete before the end of this year.	Changes at implementation site	SASG	High	Minor			
In a first approach, we have decided to involve physicians which work in cardiology and vascular surgery departments. But probably, as soon as the project develops, it will be necessary to involve more physicians from other departments because I consider we are not going to recruit the number of patients we agreed with only 2 departments. But this is not a problem.	Changes at implementation site	SASG	Medium	Minor			Assume
transfer of data to set up the decision support infrastructure	Team coordination and communication	Bio.logis	Medium	Medium			Disabled
All guideline translations must convey the same implications for the adaption of the medication. Will there be any internal validation (in addition to the efforts of the dedicated translators)?	IT / Lab infrastructure	MUWV	Low	Medium		Translations will be double-checked by each implementation site	Transfer
Regarding the CDS implementation: Mirroring the G-Standaard rules for the bio.logis rule engine might not be trivial, which could lead to delays in the implementation of the decision support infrastructure. The exact requirements of	IT / Lab infrastructure	MUWV	Medium	Severe			

the different implementation sites regarding the decision support interfaces are diverse and not always completely clear.							
Getting to the 8000 patients mark. Too early to make predictions here, but we should keep a close eye on that.	Other problems	MUWV	Low	Minor		will be monitored by WP4?	Transfer
**At the moment we have the information that the data export of rules from KNMP will not be available until end of 2016. This blocks all software development tasks at bio.logis GIM and the setup of the tool chain. Also the translations could not start.**	Team coordination and communication	Bio.logis	High	Severe		**Z-Index IT Manager needs to be contacted and a date needs to be fixed for data export (as soon as possible!)**	Avoid
Local IT infrastuctures, LIMS and EHRs and their interfaces are not presented to biologis GIM. There might arais issues. But the impact is not high because we already consider fallback mechanisms that should "simply work".	IT / Lab infrastructure	Bio.logis	Medium	Medium			
Depending on the data we finally receive from KNMP it might be that the report per patient is too big or not understandable by the doctor because he is not able to split drugs into active ingredients by himself. The GIMS is able to deal with that, but that might require further discussions.	Other problems	Bio.logis	Medium	Severe		ideally, report would contain country-specific trade names too?	

No EHR/LIMS is available. The raw PGx results provided by the genotyping platform need to be fed into the existing repository, or a new way for storing the raw results needs to be established for U-PGx. De-identification is required before PGx data can be stored. A way to automatically generate paper-based PGx reports that contain the interpreted PGx results and DPWG recommendations needs to be established. A way to generate and print the MSC cards based on the interpreted PGx results (haplotypes, phenotypes) needs to be established. Additional comment: Considering IT/Lab infrastructure challenges, we aim to identify patients eligible for PGx testing based on the first prescription of a relevant drug. Close personal contact on a daily basis and paper-based reminders will ensure that all involved physicians are aware of the relevant PGx drugs and the demands of the project. Patients will be also educated and hence, encouraged to share their PGx reports with treating physicians.	IT / Lab infrastructure	UPAT	High	Medium			
It is difficult to get staff with the appropriate expertise;	Staffing	ULMF	Low	Medium			
New staff will require extensive training	Staffing	ULMF	Medium	Medium			

**Every clinical site has different IT infrastructure, so the solutions would have to be costumized**	IT / Lab infrastructure	ULMF	Medium	Severe			Control
Political support is needed to implement IT solutions and for getting access into e-health recors and e-prescription system Additional comment: Slovenian government has started e-health record and e-prescription program. National Institute of Public Health is now coordinating these programs and is the party we need to talk to so that we could integrate U-PGx into this system. I would appreciate the support of the IT team in communication with them.	IT / Lab infrastructure	ULMF	Medium	Medium		Establish contact with responsible persons	
**The training in IT apporaches is needed for the participating clinical partners**	IT / Lab infrastructure	ULMF	Medium	Medium			
