# Supplementary material for: Pharmacogenomics decision support in the U-PGx project: Results and advice from clinical implementation across seven European countries
Source: PLoS One. 2022 Jun 8;17(6):e0268534. doi: 10.1371/journal.pone.0268534 (PMC9176797; doi:10.1371/journal.pone.0268534)
Supplement: S4 File — Questionnaire for evaluating the CDS tools among participating healthcare providers. (PDF) [file pone.0268534.s004.pdf]

ID 41

Dear participant,

Thank you for taking part in this survey. Your institution is one of the selected clinical sites participating in the multicenter study *PREPARE* of the U-PGx H2020 project that implements pre-emptive pharmacogenomic testing and evaluates its impact on therapy outcome.

For a successful implementation of pre-emptive pharmacogenomic testing, efficient decision support tools that provide you with the patients' pharmacogenomic test results and resulting drug dosing recommendations are vital. In the U-PGx project, different tools are used, depending on your institution's existing IT infrastructure and its technical capabilities. On the following pages you will be asked about your use of these tools and your opinion on them. Required questions are marked by a red asterisk. (*)

The survey takes about five to ten minutes to complete. Be assured that all answers you provide will be kept in the strictest confidentiality; only aggregate data will be reported. **Your opinion is very important to us. Your feedback will be used to make the U-PGx decision support tools the best they can be.**

Please do not hesitate to contact us if you have any questions, or if you experience any problems with the survey.

Contact

Dr.med.univ. Kathrin Blagec
kathrin.blagec@meduniwien.ac.at

Center for Medical Statistics, Informatics,

and Intelligent Systems

Medical University of Vienna

Spitalgasse 23, BT88

1090 Wien

This project has received funding from the European Union's Horizon 2020 research and innovation program under grant agreement No 668353

Institution

DATA Shortname / Alias: **Country** Variable name: **Country**

ID 9

1. What country are you located in? *

- Austria
- Greece
- Italy
- Slovenia
- Spain
- The Netherlands
- United Kingdom

LOGIC Show/hide trigger exists.
DATA Shortname / Alias: **PGx test ordered** Variable name: **PGx test ordered**
ID 12

2. **Since January 2017**, have you **at least once** ordered a pharmacogenomic test for a patient? *

- Yes
- No

DATA Shortname / Alias: **Method** Variable name: **AA ,DR ,PR ,SC ,OM**
ID 15

3. **Since January 2017**, have you **at least once** used the following methods to view or interpret a patient's pharmacogenomic test results? *

- *Please select "Digital pharmacogenomic report via the electronic health record" for all cases in which you retrieved pharmacogenomic results and recommendations via the electronic health record but **not** via an automatic alert.*
- *The **safety-code card** is a pocket card that contains an overview about the patient's pharmageonomic profile and a QR code that, after scanning, takes the user to patient-specific pharmacogenomic recommendations.*

	Yes	No
Automatic alert via the electronic health record	○	○
Digital pharmacogenomic report via the electronic health record	○	○
Paper-based pharmacogenomic report	○	○
Safety-code card	○	○
Other method(s)	○	○

(untitled)

VALIDATION Must be numeric

LOGIC Hidden unless: #2 Question "**Since January 2017**, have you **at least once** ordered a pharmacogenomic test for a patient?" is one of the following answers ("Yes")

DATA Shortname / Alias: **Number of tests** Variable name: **Number of tests**

ID 13

4. **On average**, how many pharmacogenomic tests do you order **per week**, approximately? *

(untitled)

LOGIC Hidden unless: Question "Other method(s)" is one of the following answers ("Yes")

DATA Shortname / Alias: **OM** Variable name: **OM**

ID 47

5. Please specify the **other method(s)** you used to interpret a patient's pharmacogenomic results. *

(untitled)

VALIDATION Must be numeric

LOGIC Hidden unless: Question "Automatic alert via the electronic health record" is one of the following answers ("Yes")

DATA Shortname / Alias: **AA number** Variable name: **AA number**

ID 45

6. **On average**, how often do you encounter **automatic alerts** with pharmacogenomics-based recommendations via the electronic health record **per week**, approximately? *

VALIDATION Must be numeric

LOGIC Hidden unless: Question "Digital pharmacogenomic report via the electronic health record" is one of the following answers ("Yes")

DATA Shortname / Alias: **DR_number** Variable name: **DR_number**

ID 42

7. **On average**, how many times do you use **digital pharmacogenomic reports via the electronic health record** to interpret a patient's pharmacogenomic results **per week**, approximately? *

VALIDATION Must be numeric

LOGIC Hidden unless: Question "Paper-based pharmacogenomic report" is one of the following answers ("Yes")

DATA Shortname / Alias: **PR_number** Variable name: **PR_number**

ID 43

8. **On average**, how many times do you use **paper-based pharmacogenomic reports** to interpret a patient's pharmacogenomic results **per week**, approximately? *

VALIDATION Must be numeric

LOGIC Hidden unless: Question "Safety-code card" is one of the following answers ("Yes")

DATA Shortname / Alias: **SC_number** Variable name: **SC_number**

ID 44

9. **On average**, how many times do you use the **safety-code card** to interpret a patient's pharmacogenomic results **per week**, approximately? *

VALIDATION Must be numeric

LOGIC Hidden unless: Question "Other method(s)" is one of the following answers ("Yes")

DATA Shortname / Alias: **OM_number** Variable name: **OM_number**

ID 49

10. **On average**, how many times do you use **other methods** to interpret a patient's pharmacogenomic results **per week**, approximately? *

Satisfaction with active CDS

LOGIC Hidden unless: Question "Automatic alert via the electronic health record" is one of the following answers ("Yes")

ID 84

Please rate how much you agree or disagree with the following statements about the **automatic pharmacogenomic alerts.**

LOGIC Hidden unless: Question "Automatic alert via the electronic health record" is one of the following answers ("Yes")

DATA Shortname / Alias: **AA_work_routine** Variable name: **AA_work_routine**

ID 71

11. They integrate well with my work routine. *

Strongly disagree	Disagree	Neutral	Agree	Strongly agree
○	○	○	○	○

LOGIC Hidden unless: Question "Automatic alert via the electronic health record" is one of the following answers ("Yes")

DATA Shortname / Alias: **AA_training** Variable name: **AA_training**

ID 86

12. I feel that I have received enough training to confidently use them in my daily routine. *

Strongly disagree	Disagree	Neutral	Agree	Strongly agree
○	○	○	○	○

LOGIC Hidden unless: Question "Automatic alert via the electronic health record" is one of the following answers ("Yes")

DATA Shortname / Alias: **AA_information_amount** Variable name: **AA_information_amount**

ID 111

13. **Do you feel the automatic alerts provide you with too much, too little or just the right amount of information for use in clinical practice?** *

- ○ Too little information
- ○ Just the right amount of information
- ○ Too much information

LOGIC Hidden unless: Question "Automatic alert via the electronic health record" is one of the following answers ("Yes")

DATA Shortname / Alias: **AA_satisfaction** Variable name: **AA_satisfaction**

ID 50

14. Overall, how satisfied or dissatisfied are you with the **automatic pharmacogenomic alerts**? *

Very Dissatisfied	Dissatisfied	Neutral	Satisfied	Very Satisfied
○	○	○	○	○

LOGIC Hidden unless: Question "Automatic alert via the electronic health record" is one of the following answers ("Yes")

DATA Shortname / Alias: **AA_userfriendliness** Variable name: **AA_userfriendliness**

ID 105

15. Overall, I would rate the ***user-friendliness*** of the **automatic pharmacogenomic alerts** as: *

Worst imaginable	Awful	Poor	OK	Good	Excellent	Best imaginable
○	○	○	○	○	○	○

LOGIC Hidden unless: Question "Automatic alert via the electronic health record" is one of the following answers ("Yes")

DATA Shortname / Alias: **AA_comments** Variable name: **AA_comments**

ID 51

16. Do you have any suggestions for improvement or other comments on the **automatic pharmacogenomic alerts**?

Satisfaction with passive CDS

LOGIC Hidden unless: Question "Digital pharmacogenomic report via the electronic health record" is one of the following answers ("Yes")

ID 82

Please rate how much you agree or disagree with the following statements about the digital pharmacogenomic reports in the electronic health record:

LOGIC Hidden unless: Question "Digital pharmacogenomic report via the electronic health record" is one of the following answers ("Yes")

DATA Shortname / Alias: **DR_work_routine** Variable name: **DR_work_routine**

ID 72

17. They integrate well with my work routine. *

Strongly disagree	Disagree	Neutral	Agree	Strongly agree
○	○	○	○	○

LOGIC Hidden unless: Question "Digital pharmacogenomic report via the electronic health record" is one of the following answers ("Yes")

DATA Shortname / Alias: **DR_training** Variable name: **DR_training**

ID 83

18. I feel that I have received enough training to confidently use them in my daily routine. *

Strongly disagree	Disagree	Neutral	Agree	Strongly agree
○	○	○	○	○

LOGIC Hidden unless: Question "Digital pharmacogenomic report via the electronic health record" is one of the following answers ("Yes")

DATA Shortname / Alias: **DR_training** Variable name: **DR_training**

ID 112

19. **Do you feel the <u>digital pharmacogenomic reports</u> provide you with too much, too little or just the right amount of information for use in clinical practice?** *

- ○ Too little information
- ○ Just the right amount of information
- ○ Too much information

LOGIC Hidden unless: Question "Digital pharmacogenomic report via the electronic health record" is one of the following answers ("Yes")

DATA Shortname / Alias: **DR_satisfaction** Variable name: **DR_satisfaction**

ID 53

20. Overall, how satisfied or dissatisfied are you with the **digital pharmacogenomic reports in the electronic health record**? *

Very Dissatisfied	Dissatisfied	Neutral	Satisfied	Very Satisfied
○	○	○	○	○

LOGIC Hidden unless: Question "Digital pharmacogenomic report via the electronic health record" is one of the following answers ("Yes")

DATA Shortname / Alias: **DR_userfriendliness** Variable name: **DR_userfriendliness**

ID 107

21. Overall, I would rate the ***user-friendliness*** of the **digital pharmacogenomic reports** as: *

Worst imaginable	Awful	Poor	OK	Good	Excellent	Best imaginable
○	○	○	○	○	○	○

LOGIC Hidden unless: Question "Digital pharmacogenomic report via the electronic health record" is one of the following answers ("Yes")

DATA Shortname / Alias: **DR_comments** Variable name: **DR_comments**

ID 54

22. Do you have any suggestions for improvement or other comments on the **digital pharmacogenomic reports in the electronic health record**?

How do you like the structure of the report? Is the report too long? Is there any information that could be omitted in your opinion? Is there any information missing? Any other ideas for improvement?

Satisfaction with paper-based PGx reports

LOGIC Hidden unless: Question "Paper-based pharmacogenomic report" is one of the following answers ("Yes")

ID 80

Please rate how much you agree or disagree with the following statements about the paper-based pharmacogenomic reports:

LOGIC Hidden unless: Question "Paper-based pharmacogenomic report" is one of the following answers ("Yes")

DATA Shortname / Alias: **PR_workroutine** Variable name: **PR_workroutine**

ID 73

23. They integrate well with my work routine. *

Strongly disagree	Disagree	Neutral	Agree	Strongly agree
○	○	○	○	○

LOGIC Hidden unless: Question "Paper-based pharmacogenomic report" is one of the following answers ("Yes")

DATA Shortname / Alias: **PR_training** Variable name: **PR_training**

ID 81

24. I feel that I have received enough training to confidently use them in my daily routine. *

Strongly disagree	Disagree	Neutral	Agree	Strongly agree
○	○	○	○	○

LOGIC Hidden unless: Question "Paper-based pharmacogenomic report" is one of the following answers ("Yes")

DATA Shortname / Alias: **PR_information** Variable name: **PR_information**

ID 113

25. **Do you feel the <u>paper-based pharmacogenomic reports</u> provide you with too much, too little or just the right amount of information for use in clinical practice?** *

- ○ Too little information
- ○ Just the right amount of information
- ○ Too much information

LOGIC Hidden unless: Question "Paper-based pharmacogenomic report" is one of the following answers ("Yes")

DATA Shortname / Alias: **PR_satisfaction** Variable name: **PR_satisfaction**

ID 56

26. Overall, how satisfied or dissatisfied are you with the **<u>paper-based pharmacogenomic reports</u>**? *

Very Dissatisfied	Dissatisfied	Neutral	Satisfied	Very Satisfied
○	○	○	○	○

LOGIC Hidden unless: Question "Paper-based pharmacogenomic report" is one of the following answers ("Yes")

DATA Shortname / Alias: **PR_userfriendliness** Variable name: **PR_userfriendliness**

ID 108

27. Overall, I would rate the ***user-friendliness*** of the **paper-based pharmacogenomic reports** as: *

Worst imaginable	Awful	Poor	OK	Good	Excellent	Best imaginable
○	○	○	○	○	○	○

LOGIC Hidden unless: Question "Paper-based pharmacogenomic report" is one of the following answers ("Yes")

DATA Shortname / Alias: **PR_comments** Variable name: **PR_comments**

ID 57

28. Do you have any suggestions for improvement or other comments on the **paper-based pharmacogenomic reports**?

How do you like the structure of the report? Is the report too long? Is there any information that could be omitted in your opinion? Is there any information missing? Any other ideas for improvement?

Satisfaction with MSC System I

LOGIC Hidden unless: Question "Safety-code card" is one of the following answers ("Yes")

ID 79

Please rate how much you agree or disagree with the following statements:

LOGIC Hidden unless: Question "Safety-code card" is one of the following answers ("Yes")
DATA Shortname / Alias: **SC_workroutine** Variable name: **SC_workroutine**
ID 74

29. Using the **safety-code card** integrates well with my work routine. *

Strongly disagree	Disagree	Neutral	Agree	Strongly agree
○	○	○	○	○

LOGIC Hidden unless: Question "Safety-code card" is one of the following answers ("Yes")
DATA Shortname / Alias: **SC_training** Variable name: **SC_training**
ID 78

30. I feel that I have received enough training to confidently use the **safety-code card** in my daily routine. *

Strongly disagree	Disagree	Neutral	Agree	Strongly agree
○	○	○	○	○

LOGIC Hidden unless: Question "Safety-code card" is one of the following answers ("Yes")
DATA Shortname / Alias: **SC_information** Variable name: **SC_information**
ID 114

31. **Do you feel the safety-code card provides you with too much, too little or just the right amount of information for use in clinical practice?** *

- ○ Too little information
- ○ Just the right amount of information
- ○ Too much information

LOGIC Hidden unless: Question "Safety-code card" is one of the following answers ("Yes")

DATA Shortname / Alias: **SC_satisfaction** Variable name: **SC_satisfaction**

ID 59

32. Overall, how satisfied or dissatisfied are you with the **safety-code card?**
*

Very Dissatisfied	Dissatisfied	Neutral	Satisfied	Very Satisfied
○	○	○	○	○

LOGIC Hidden unless: Question "Safety-code card" is one of the following answers ("Yes")

DATA Shortname / Alias: **SC_userfriendliness** Variable name: **SC_userfriendliness**

ID 109

33. Overall, I would rate the ***user-friendliness*** of the **safety-code card** as: *

Worst imaginable	Awful	Poor	OK	Good	Excellent	Best imaginable
○	○	○	○	○	○	○

LOGIC Hidden unless: Question "Safety-code card" is one of the following answers ("Yes")

DATA Shortname / Alias: **SC_comments** Variable name: **SC_comments**

ID 60

34. Do you have any suggestions for improvement or other comments on the **safety-code card?**

Do you think the safety-code card is a useful tool? Why, why not? Do you have any comments on the website that displays the dosing recommendations? Any ideas for improvement?

(untitled)

ID 87

Please rate how much you agree or disagree with the following statement:

DATA Shortname / Alias: **General_enough_training** Variable name: **General_enough_training**

ID 75

35. I feel that I have received enough training to confidently use pharmacogenomic test results in my daily routine. *

Strongly disagree	Disagree	Neutral	Agree	Strongly agree
○	○	○	○	○

LOGIC Hidden unless: (((Question "Automatic alert via the electronic health record" is one of the following answers ("Yes") OR Question "Digital pharmacogenomic report via the electronic health record" is one of the following answers ("Yes")) OR Question "Paper-based pharmacogenomic report" is one of the following answers ("Yes")) OR Question "Safety-code card" is one of the following answers ("Yes"))

DATA Shortname / Alias: **General_comments** Variable name: **General_comments**

ID 67

36. Do you have any other comments on the U-PGx decision support tools, the pharmacogenomic dosing recommendations or is there anything else you would like us to know?

Demographics I

ID 68

Thank you! You are almost done. Finally, we would like to ask you a few demographic questions.

DATA Shortname / Alias: **Gender** Variable name: **Gender**

ID 7

37. Are you male or female?

- Male
- Female
- Prefer not to state

DATA Shortname / Alias: **Age** Variable name: **Age**

ID 100

38. How old are you?

DATA Shortname / Alias: **Years_experience** Variable name: **Years_experience**

ID 101

39. How many years of experience do you have in your profession?

DATA Shortname / Alias: **Years_experience_PGx** Variable name: **Years_experience_PGx**

ID 115

40. How many years of experience do you have with pharmacogenomics-guided prescribing (approximately)?

In case you have less than one year experience, you can enter a fraction (e.g., 0.5 for a half year).

[ ]

DATA Shortname / Alias: **Field_of_work**

ID 97

41. Which of the following best describes your field of work?

- ☐ Anesthesiology
- ☐ Clinical pharmacology
- ☐ Emergency medicine
- ☐ Internal medicine
- ☐ Nephrology
- ☐ Neurology
- ☐ Obstetrics / Gynaecology
- ☐ Oncology
- ☐ Pediatrics
- ☐ Pharmacy
- ☐ Primary care / Family medicine
- ☐ Psychiatry
- ☐ Rheumatology
- ☐ Surgery
- ☐ Other - please specify

[ ]

DATA Shortname / Alias: **Treatment_setting** Variable name: **Treatment_setting**

ID 104

42. Most of the time, are you dealing with patients in an inpatient or outpatient setting?

- Inpatient setting
- Outpatient setting
- Half inpatient / half outpatient setting

ID 95

Please rate how much you agree or disagree with the following statements:

DATA Shortname / Alias: **Comfortable_computers** Variable name: **Comfortable_computers**

ID 93

43. I am comfortable using computers.

Strongly disagree	Disagree	Neutral	Agree	Strongly agree
○	○	○	○	○

DATA Shortname / Alias: **Comfortable_mobile_devices** Variable name: **Comfortable_mobile_devices**

ID 102

44. I am comfortable using mobile devices such as smartphones or tablets.

Strongly disagree	Disagree	Neutral	Agree	Strongly agree
○	○	○	○	○

DATA Shortname / Alias: **Comfortable_EHR** Variable name: **Comfortable_EHR**

ID 94

45. I am comfortable using electronic health records.

Strongly disagree	Disagree	Neutral	Agree	Strongly agree
○	○	○	○	○

Thank You!

ID 1

Thank you very much for your time, we appreciate your feedback! You are helping us to improve the U-PGx decision support tools.
